# Outcome of 5-Year Treatment of Neovascular Age-Related Macular Degeneration With Intravitreal Anti-VEGF Using “Treat and Extend” Regimen

**DOI:** 10.3389/fmed.2018.00125

**Published:** 2018-05-01

**Authors:** Polona Jaki Mekjavic, Polona Zaletel Benda

**Affiliations:** ^1^Eye Hospital, University Medical Center Ljubljana, Ljubljana, Slovenia; ^2^Medical Faculty, University of Ljubljana, Ljubljana, Slovenia

**Keywords:** treat and extend regimen, anti-VEGF agent, neovascular age-related macular degeneration, 5 years follow-up, long-term visual acuity outcome

## Abstract

**Objective:**

The aim of this study is twofold. *First*, to evaluate the long-term outcome of anti-vascular endothelial growth factor (anti-VEGF) treatment in a clinical setting using the “treat-and-extend regimen” (TER) in patients with neovascular age-related macular degeneration (nAMD). *Second*, to determine the proportion of patients treated with anti-VEGF with good visual acuity (VA), i.e., vision sufficient to maintain a high level of independence.

**Design:**

We conducted a single center retrospective review of patients with treatment-naive nAMD who were treated with anti-VEGF. Patients were treated with anti-VEGF intravitreal injections according to the TER. Patients started treatment with monthly injections of either bevacizumab (1.25 mg/0.05 mL) or ranibizumab (0.5 mg/0.05 mL) until there were no signs present of choroidal neovascularization (CNV) activity. CNV activity was determined from fundus examination and SD-OCT imaging. Follow-up administration of intravitreal injections was extended by 2-week intervals, up to a total of 14 weeks, provided no signs of CNV activity were detected. In some patients, the first treatment was replaced with aflibercept (2 mg/0.05 mL).

**Participants:**

On the basis of the inclusion criterion for the study, that patients had to be treated for 5 years, a total of 101 patients were included in the study. In all patients, one eye was treated for a 5-year period, and thus we studied 101 eyes.

**Measurements:**

Best corrected VA was analyzed at baseline and each year during the 5-year follow-up.

**Results:**

VA improved initially after year 1 of the treatment. VA decreased in the subsequent 4 years of treatment, but remained significantly higher from year 1 to year 3 of the treatment compared to baseline values. Patients with good VA followed a similar trend: the proportion increased in the first year, and thereafter gradually decreased during the course of the 5-year follow up. At year 5, the number of patients with good VA decreased to baseline values.

**Conclusion:**

TER with anti-VEGF for nAMD treatment prevents long-term severe visual loss in real-world setting and maintains patients’ VA at levels sufficient to ensure independence.

## Introduction

According to World Health Organization, age-related macular degeneration (AMD) is the third cause of blindness globally and a primary cause of blindness in developed countries, particularly in older adults (1). In their systematic review, Wong et al. reported that 8.7% of the world adult population in the age group of 45–85 years had AMD (2). According to a meta-analysis of AMD prevalence in Europe, the prevalence of AMD increased exponentially with age (odds ratio 4.2 per decade) (3). It is predicted that the global incidence of AMD will increase progressively from 196 million in 2020 to 288 million in 2040, due to increased life expectancy, and the negative impact of environmental factors (2). AMD is a disease that affects central vision needed for daily activities such as reading, driving, and facial recognition leading to greater social dependence. With reduced central vision, people cannot fulfill the criteria for a driving license (4) and have difficulty reading (5). Decreased independence leads to higher rates of clinical depression, increased risk of falls and premature admission to nursing homes (6). Late AMD can be atrophic or neovascular, and the estimated prevalence of late AMD is 1.4% at 70 years of age, increasing to 5.6% at age 80 years and 20% at age 90 years (3). Worldwide projections have shown a doubling of late AMD by 2040 (2). Severe vision loss occurs in up to 80% of AMD cases (7), the majority of them have the neovascular form of the illness. The neovascular form of AMD (nAMD) is caused by the growth of new blood vessels termed choroidal neovascularization (CNV) and causes a rapid deterioration of vision. If nAMD is left untreated, severe vision loss (≥15 ETDRS letters) occurs within 2 years (8).

Evidence indicates the pivotal role of vascular endothelial growth factor (VEGF) in nAMD development. VEGF in the eye is largely produced by retinal pigment epithelial cells. In response to heterogeneous stressors, the level of VEGF in vitreous increases and can result in pathological conditions such as nAMD. VEGF influences initial stages of CNV formation by promoting angiogenesis and vasculogenesis. VEGF also increases vascular permeability, because it disrupts the intracellular junction of vascular endothelial cells and induces formation of pores in the vessel wall (9, 10). Since VEGF plays a pivotal role in the pathogenesis of nAMD, it has become the main target for the treatment. Different anti-VEGF molecules have been developed and three are currently used in clinical practice: ranibizumab, bevacizumab, and aflibercept. Anti-VEGF therapy with intravitreal injections is the current standard therapy for nAMD. The goal of anti-VEGF treatment is to minimize vision loss and disability in order to maintain independence. Anti-VEGF therapy has dramatically reduced the incidence of legal blindness caused by nAMD (11–13).

In 2006, key randomized clinical trials, MARINA and ANCHOR, introduced ranibizumab for nAMD treatment, the therapy comprising monthly intravitreal injections (8, 14). From a socioeconomic point of view, this regimen of monthly injections of anti-VEGF agent raised concerns that the treatment was not cost-effective. The first individualized treatment regimen introduced was the “as-needed” or *pro re nata* (PRN) regimen that required monthly optical coherent tomography (OCT) imaging and clinical examination to detect early disease recurrence. It also guided the need for retreatment. The patient received an injection only, if the OCT showed a recurrence of fluid or hemorrhage. Thus, the PRN regimen allows for a reduced number of injections, but monthly assessment visits are required (15). The “treat-and-extend” regimen (TER) aims to improve the PRN treatment by reducing the burden of frequent treatments, specifically the number of visits and injections. The TER regimen provides an individualized dosing regimen (16). TER consists of a loading dose of initial monthly injections. Once visual acuity (VA) is stable, macular hemorrhage is absent, and no excessive retinal hydration was revealed on OCT, patients continue to receive regular maintenance injections. At that point, the interval between treatments increases, 2 weeks at a time, as long as the retina remains dry and stable, up to a 14-week interval. At each visit, best corrected VA, clinical findings, and OCT changes are recorded, and patients receive an injection regardless of the presence or absence of disease activity. If there are no changes in the above parameters, the interval to the next visit is extended for 2 weeks. However, if there is evidence of disease activity, the interval for the next scheduled injection and examination is shortened by 2 weeks (17). According to the 2015 Global Trends in Retina Survey published by the American Society of Retina Specialist, TER was the most commonly applied regimen of anti-VEGF treatment for nAMD in the United States. 66.2% retina specialist preferred TER to other types of treatment regimens (16).

In clinical practice under real-world conditions (i.e., not conducted within the framework of a clinical study; results are obtained from clinical practice), different results are expected compared to well-conducted tightly controlled prospective clinical trials, due to different conditions in which these studies are conducted. In the Comparison of Age-Related Macular Degeneration Treatments Trials Follow-up Study (CATT follow-up study), patients were carefully selected and treated for 2 years in a well-controlled study protocol. After this 2-year period, patients were treated in real-world conditions for 3 years. During the proceeding 3-year period, mean VA declined by 3 letters compared to baseline and by 11 letters compared to the values at the end of the initial 2-year period (18). Meta-analysis of real-world outcomes of intravitreal ranibizumab for the treatment of nAMD reported the greatest improvement in VA in the first year of treatment. VA gradually declined in the second and subsequent years of treatment. By contrast, a recent report of long-term vision outcome of the VEGF Trap-Eye.

Investigation of Efficacy and Safety in Wet AMD 1 Extension Study indicates that treatment with aflibercept with fixed-interval dosing maintained visual improvement at 4 years (19). TER demonstrated better visual outcomes in comparison to the PRN regimen of anti-VEGF therapy (20). Recently, several retrospective studies evaluated the efficacy of the TER regimen of anti-VEGF therapy under real-world conditions (21–23). Whereas the studies by Pedrosa et al. (21) and Gillies et al. (22) included different treatment regimens in their analysis, the study by Mrejen et al. (23) evaluated only the TER regimen, but followed only 54 patients for 5 years. More studies using only one treatment regimen for a prolonged period of time are essential to provide assessment of the efficacy of anti-VEGF intravitreal injections in patients with nAMD and thus to improve treatment recommendations.

The primary aim of our study was to analyze the long-term visual outcomes of anti-VEGF treatment using TER in patients with nAMD treated for 5 years in a routine clinical practice under real-world conditions in the only tertiary eye center in Slovenia. The secondary aim of this study was to determine the proportion of treated patients who retained good vision, defined as vision sufficient for maintaining a high level of independence (i.e. including driving and reading ability).

## Materials and Methods

### Patient Selection

This study used a retrospective collection of data from patients who were treated with intravitreal therapy after being newly diagnosed with nAMD. The patients were treated as part of routine clinical practice at the only academic retinal practice in Slovenia (Eye Hospital, University Medical Center Ljubljana). The study included all patients who were continuously treated at the Eye Hospital for at least 5 years and completed their 5-year follow-up in the period from July 2013 to September 2016. We only included one eye of each patient as no patient had a 5-year follow-up of treatment in both eyes in the mentioned period. During each visit, we measured the patients best-corrected VA with ETDRS charts, performed SD-OCT (Topcon 3D OCT 1000) and funduscopic examinations. Patients received anti-VEGF injections on the same day. All patients whose data were collected gave written consent for the use of their anonymized data for clinical audit and research purposes. The study was approved by the National Medical Ethics Committee at the Ministry of Health (Republic of Slovenia) and complied with the ethical principles set by the Declaration of Helsinki.

All patients included in the analysis had active and treatment naive nAMD, i.e., never received any form of treatment for nAMD. Patients were diagnosed for active nAMD on the basis of clinical examination, fluorescein angiography (FA), and SD-OCT. Exclusion criteria included: VA at time of diagnosis less then 20/200 Snellen equivalent; CNV due to any other retinal diseases such as myopic, post-inflammation, or idiopathic CNV; any concomitant retinal disease such as diabetic retinopathy or vein occlusion; previous treatment of CNV in the study eye, such as photodynamic therapy and/or laser photocoagulation. Patients who submitted to cataract surgery during the follow-p were not excluded.

### Treatment Regimen

Patients were treated with anti-VEGF intravitreal injections according to the TER. The patients started treatment with either bevacizumab (1.25 mg/0.05 mL; Avastin; Genentech, San Francisco, CA, USA) or ranibizumab (0.5 mg/0.05 mL; Lucentis; Genentech, San Francisco, CA, USA) and received at least three monthly intravitreal injections until there were no further signs of CNV activity, as determined with fundus examination and SD-OCT imaging. Thereafter, the follow-ups and administration of intravitreal injections were extended by 2-week interval, for as long as no signs of CNV activity were detected, up to a total of 14 weeks. Patients received an injection on each visit. If there was any sign of CNV activity, such as new hemorrhage and increase or persistence of intra- or sub-retinal fluid, despite consecutive injections, follow-up intervals were shortened by 2 weeks. Some of the patients switched to anti-VEGF aflibercept (2.0 mg/0.05 mL; Eylea; Bayer Healthcare, Berlin, Germany) due to changed reimbursement policy or sometimes suboptimal response to treatment as determined by the treating physician.

### Data Collection

Data collected from patients records were age at the onset of the treatment, gender, and VA. VA of studied eyes, determined by ETDRS charts, was collected from documentation at 6 time points: at baseline and after every year of treatment (±6 weeks): after first, second, third, fourth, and fifth year of follow-up. The number of anti-VEGF injections received in each year of treatment and type of anti-VEGF agent was recorded.

For the present analysis, patients were divided into three groups according to their VA: (i) good VA: VA ≥ 65 ETDRS letters (Snellen equivalent 20/40); (ii) intermediate VA: VA between 36–64 ETDRS letters; (iii) low VA: VA ≤ 35 ETDRS letters. The proportion of patients with good VA ≥ 65 ETDRS was calculated for each year of treatment. A VA of 65 ETDRS is 20/40 or 0.5 Snellen equivalent, considered the threshold for reading (5) and driving (4) ability.

Morphological characteristics of nAMD lesion of patients losing ≥15 ETDRS letters after 5 years were determined by analyzing the images of FA during the baseline visit and SD-OCT and color fundus picture during at each visit.

### Statistical Analysis

The change in VA and the number of injections were calculated using descriptive statistics (mean, SD of mean, range), and the mean in three subgroups of patients compared with ANOVA analysis. A *post hoc* test (Tukey’s Honest Significant Difference method) was used to compare mean data points between the subgroups. The two-tailed *t*-test for independent samples was used to compare mean data points in two groups of patients. Categorical variables were reported as proportions. Statistical calculations were performed with SPSS software version 22 (SPSS, Inc., Chicago, IL, USA). In all comparisons of means, the level of significance was set at 0.05.

## Results

### Baseline Characteristics

A total of 101 eyes of 101 patients (76 females, 25 males) with treatment naive nAMD, who were all followed for 5 years, were enrolled in this study. The mean age of all patients at the start of treatment was 81.8 ± 6.4 years (range: 66–97 years). The mean age of the female patients was 82.3 ± 5.7 years (range: 69–97 years) and of the male patients was 80.3 ± 8.0 years (range 66–95 years). Baseline mean VA was 60.5 ± 11.3 ETDRS letters (range 30 – 85 ETDRS letters) (Table 1).

**Table 1:** Baseline characteristics of the study group.

Parameter	Study group
Patients (*N*)	101
Eyes (*N*)	101
Age at diagnosis (years), mean ± SD, range	81 ± 6.4, 66–97
Sex (*N*); male/female	25/76
Baseline VA (ETDRS letters); mean ± SD, range	60.5 ± 11.3, 30–85

*N, number; VA, best corrected visual acuity; ETDRS, Early Treatment Diabetic Retinopathy Study*.

### VA Outcomes

**Figure 1:**
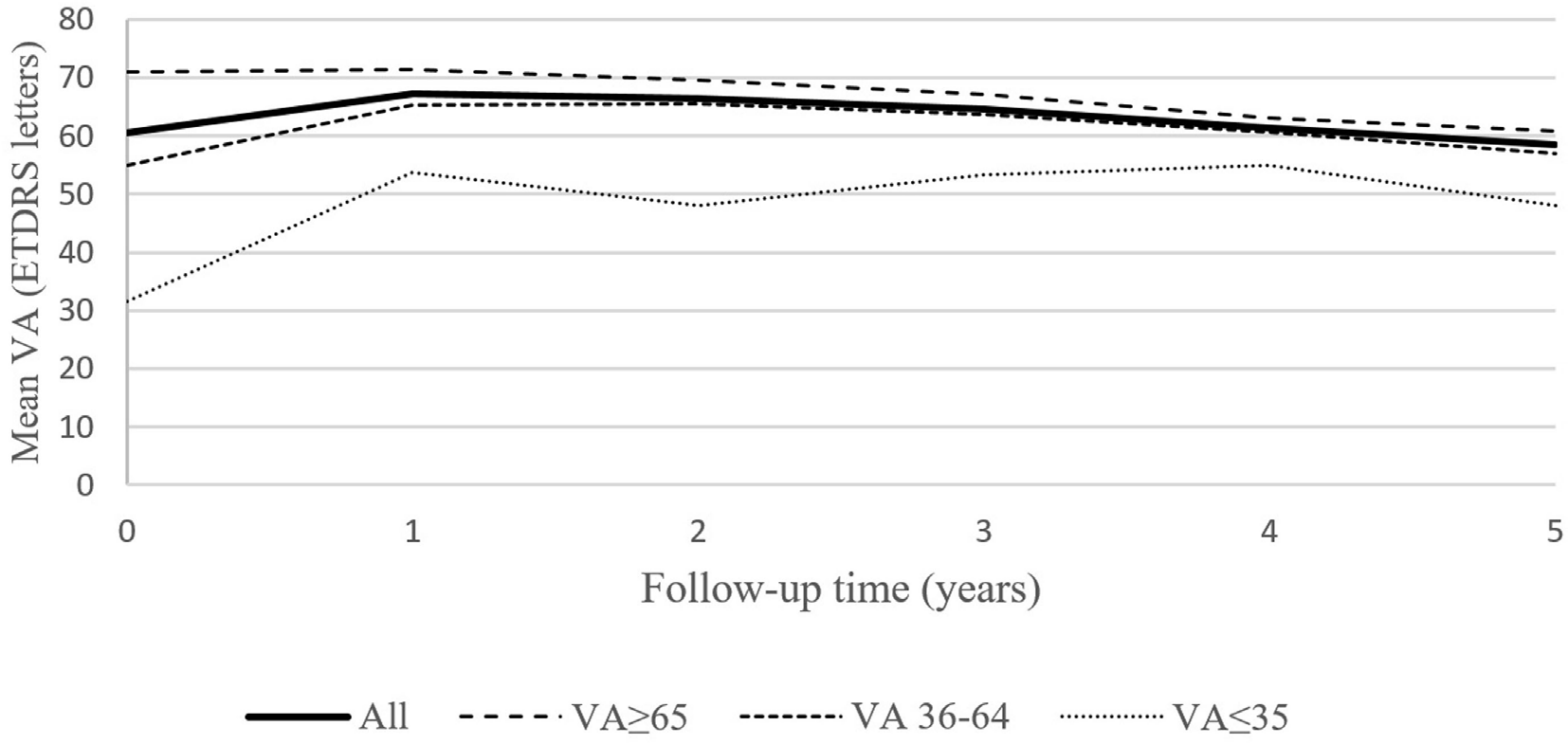
Mean best corrected visual acuity (VA) for the entire group of 101 patient, and for the subgroups of patients based on baseline VA (40 patients with baseline VA ≥ 65 letters, 57 patients with baseline VA = 36–64 and 4 patients with baseline VA ≤ 35 letters) during the 5-year follow-up.

Mean VA at the final follow-up visit after 5 years of treatment was 58.6 (± 18.3) letters, which was not significantly different (p > 0.05) from the values observed at the onset of treatment (60.5 ± 11.3). During the first three years of treatment there was an overall improvement in VA. Specifically, the significant improvement (p < 0.05) of VA after the first year was retained in the subsequent 2 years of treatment (Table 2; Figure 1), despite a tendency of a progressive decrease. VA after four and five years of treatment was not statistically different from baseline VA.

**Table 2:** VA, change of VA compared to baseline and number of intravitreal injections for 101 patients and for subgroups of those who avoided and those who did not avoid significant loss (≥15 ETDRS letters) of vision during the 5-year follow-up.

	All patients			Patients avoiding loss ≥ 15 letters			Patients with loss ≥ 15 letters		
No. of patients	101			81			20		
Age (years ± SD)	81.8 ± 6.4			81.5 ± 6.5			83.1 ± 5.9		
Gender (no. of F/M)		76/25			64/17			12/8	

**Years of treatment**	**VA, mean ± SD (ETDRS letters)**	**VA change (ETDRS letters)**	**Injections, mean ± SD (number)**	**VA, mean ± SD (ETDRS letters)**	**VA change (ETDRS letters)**	**Injections, mean ± SD (number)**	**VA, mean ± SD (ETDRS letters)**	**VA change (ETDRS letters)**	**Injections, mean ± SD (number)**

Baseline	60.5 ± 11.3	/	/	59.2 ± 11.5•	/	/	65.7 ± 8.8•	/	/
First year	67.3 ± 10.6*	+6.8	8.0 ± 1.3	67.5 ± 11.1*	+8.3	8.0 ± 1.2	66.3 ± 8.9	+0.6	8.1 ± 1.4
Second year	66.4 ± 11.7*	+5.9	6.3 ± 1.9	67.0 ± 11.9*	+7.8	6.3 ± 1.8	64.0 ± 10.2	−1.7	6.2 ± 2.5
Third year	64.5 ± 11.7*	+4.0	5.9 ± 1.9	65.2 ± 11.9*	+6.0	5.9 ± 1.9	61.8 ± 10.6	−3.9	6.2 ± 2.0
Fourth year	61.4 ± 13.0	+0.9	5.4 ± 1.8	63.9 ± 12.0*	+4.7	5.5 ± 1.8	51.3 ± 12.2*	−14.4	5.1 ± 1.4
Fifth year	58.6 ± 18.3	−1.9	4.0 ± 1.8	63.1 ± 11.4*	+3.9	5.0 ± 1.7	50.4 ± 11.2*	−15.3	5.1 ± 2.3
In all 5 years			30.5 ± 5.5			30.6 ± 5.4			33.4 ± 5.9

*VA, best corrected visual acuity; ETDRS, Early Treatment Diabetic Retinopathy Study; F/M, female/male*.

*p-value < 0.05 compared to baseline.

*^•^p-value < 0.05 between the subgroups*.

Patients were divided into three groups according to their baseline VA (Table 3): (i) “good baseline VA” group: defined as VA ≥ 65 ETDRS letters (*N* = 40); (ii) “intermediate baseline VA” group: defined as VA between 36–64 ETDRS letters (*N* = 57); (iii) “low baseline VA” group: defined as VA ≤ 35 ETDRS letters (*N* = 4). Subgroups were homogeneous for age (*p* = 0.32). During 5 years of treatment, patients in the “good baseline VA” group gained less letters or lost more letters compared to the other two groups. Patients in the “intermediate baseline VA” group had statistically significant improvement in VA in the first 4 years of treatment. The group of patients with “low baseline VA gained the most letters, but the gain in VA was not statistically significant. This may also be attributed to a small sample size.

**Table 3:** VA, change of VA compared to baseline, and number of intravitreal injections during the 5 years of treatment for patients assigned to groups according to their VA at the beginning of treatment: “good baseline VA” group with baseline VA ≥ 65 ETDRS letters, “intermediate baseline VA” group with baseline VA 36–64 ETDRS letters and “low baseline VA” group with baseline VA ≤ 35 ETDRS letters.

	Good baseline VA			Intermediate baseline VA			Low baseline VA		
No. of patients	40			57			4		
Age (years ± SD)	81.5 ± 6.7			82.4 ± 6.0			77.5 ± 7.4		
Gender (no. of F/M)	26/14			48/9			2/2		

**Years of treatment**	**VA, Mean ± SD (ETDRS letters)**	**VA change (ETDRS letters)**	**Injections, Mean ± SD (number)**	**VA, Mean ± SD (ETDRS letters)**	**VA change (ETDRS letters)**	**Injections, Mean ± SD (number)**	**VA, Mean ± SD (ETDRS letters)**	**VA change (ETDRS letters)**	**Injections, Mean ± SD (number)**

Baseline	71.1 ± 11.3	/	/	55.0 ± 6.2	/	/	31.5 ± 2.2	/	/
First year	71.4 ± 9.1	+0.3	8.2 ± 1.2	65.4 ± 9.8*	+10.4	7.9 ± 1.3	53.8 ± 16.3	+22.3	7.5 ± 0.5
Second year	69.5 ± 9.6	−1.6	6.6 ± 2.0	65.5 ± 11.4*	+10.5	6.2 ± 1.8	48.0 ± 15.5	+16.5	4.3 ± 1.3
Third year	67.1 ± 10.2*	−4.0	6.1 ± 1.8	63.6 ± 11.9*	+8.6	5.8 ± 1.9	53.3 ± 14.2	+21.8	5.3 ± 1.1
Fourth year	63.1 ± 13.7*	−8.0	5.4 ± 1.9	60.6 ± 12.0*	+5.6	5.4 ± 1.6	55.0 ± 15.9	+23.5	5.5 ± 2.0
Fifth year	60.9 ± 16.2*	−10.2	5.2 ± 1.9	57.0 ± 13.0	+2.0	4.8 ± 1.7	48.0 ± 12.8	+16.5	5.7 ± 1.7
In all 5 years			31.5 ± 5.7			30.1 ± 5.3			27.3 ± 4.8

*VA, best corrected visual acuity; ETDRS, Early Treatment Diabetic Retinopathy Study; F/M, female/male*.

*p-value < 0.05 compared to baseline.

It is necessary to emphasize that the final VA after 5 years of treatment was still better in the “good baseline VA” group of patients compared to the remaining two groups (Figure 1). The difference in the number of injections between the good and intermediate baseline VA groups was not statistically significant.

After 5 years of treatment, the patients were again assigned to three groups based on their VA after 5 years of treatment (Table 4): “good final VA” group with VA > 65 letters (*N* = 39), “intermediate final VA” group with VA between 36–64 ETDRS letters (*N* = 54), and “low final VA” group with VA ≤ 35 ETDRS letters (*N* = 8). There was no statistically significant difference in the age distribution between the three groups. During the 5 years of treatment, the “good final VA” group maintained significantly better VA compared to their baseline mean VA. Patients in the “intermediate final VA” group maintained a statistically significant gain for 2 years. The difference in the number of injections received by patients in the “good final VA” group and “intermediate final VA” group was not statistically significant. In the “low final VA” group that consisted of eight patients, a statistically significant gain in VA was not observed. In this group, after 5 years of treatment, there was a statistically significant decrease in VA, and the number of injections received was significantly lower. Baseline mean VA in the “good final VA” group was significantly better compared to the “intermediate final VA group” (*p* = 0.008). The difference in baseline mean VA was not statistically significant between these two groups and the “low final VA group,” most probably due to the small sample size of the latter.

**Table 4:** VA, change of VA compared to baseline and number of intravitreal injections during the 5 years of treatment for patients assigned to groups according to their VA after 5 years of treatment: “good final VA” group with final VA ≥ 65 ETDRS letters, “intermediate final VA” group with final VA 36–64 ETDRS letters, and “low final VA” group with final VA ≤ 35 ETDRS letters.

	Good final VA			Intermediate final VA			Low final VA		
No. of patients	39			54			8		
Age (years ± SD)	80.8 ± 6.6			83.0 ± 6.4			79.1 ± 2.9		
Gender (no. of F/M)	28/10			42/12			6/2		

	**VA, mean ± SD(ETDRS letters)**	**VA variation (ETDRS letters)**	**Injections, number ± SD**	**VA, mean± SD (ETDRS letters)**	**VA variation (ETDRS letters)**	**Injections, number ± SD**	**VA, mean ± SD (ETDRS letters)**	**VA variation (ETDRS letters)**	**Injections, number ± SD**

Baseline	65.8 ± 8.8•	/	/	57.9 ± 10.7•	/	/	56.8 ± 13.3	/	/
First year	74.0 ± 6.9*	+8.2	7.9 ± 1.3	62.6 ± 10.5*	+4.7	8.1 ± 1.2	65.1 ± 8.3	+8.3	7.5 ± 1.1
Second year	74.3 ± 5.6*	+8.5	6.2 ± 1.8	61.3 ± 11.4*	+3.4	6.5 ± 1.9	62.3 ± 14.2	+5.5	4.6 ± 2.3
Third year	72.3 ± 6.4*	+6.5	5.9 ± 1.6	59.5 ± 11.4	+1.6	6.0 ± 2.1	60.4 ± 13.0	+3.6	5.3 ± 1.3
Forth year	71.6 ± 6.0*	+5.8	5.3 ± 2.0	56.1 ± 11.8	−1.8	5.6 ± 1.7	46.4 ± 9.2	−10.4	4.1 ± 0.8
Fifth year	72.5 ± 5.3*	+6.7	5.3 ± 1.6	52.8 ± 8.1*	−5.1	5.0 ± 1.8	28.1 ± 5.0	−28.7*	3.6 ± 2.1
In all 5 years			30.5 ± 5.4			31.4 ± 5.4			25.1 ± 3.7

*VA, best corrected visual acuity; ETDRS, Early Treatment Diabetic Retinopathy Study; F/M, female/male*.

*p-value < 0.05 compared to baseline.

*^•^p-value < 0.05 between the subgroups*.

At baseline, 40% of patients had good VA. After the first year, this proportion increased to 67%, but it decreased gradually in subsequent years. The proportion of patients with intermediate VA remained stable. The proportion of patients with low VA decreased in the first year from 4% to 0%, but gradually increased in subsequent years (Table 5).

**Table 5:** Number of the patients (*n* = 101) divided into groups according to VA in each group of 5-year follow-up: good VA (VA ≥ 65 ETDRS letters), intermediate VA (VA 36–64 ETDRS letters), low VA (≤35 ETDRS letters); patients that avoided visual loss ≥ 15 ETDRS letters, that gained ≥ 15 ETDRS letters, that had stable VA (variation ± 15 ETDRS letters) and that lost ≥ 15 letters.

No. of patients	Baseline	First year	Second year	Third year	Fourth year	Fifth year
Good VA (ETDRS letters)	40	67	65	61	52	39
Intermediate VA (ETDRS letters)	57	34	34	37	44	54
Low VA (ETDRS letters)	4	0	2	3	5	8
Avoiding loss ≥ 15 ETDRS letters	/	100	97	94	86	81
Gaining ≥ 15 ETDRS letters	/	19	26	26	14	10
Variation of 15 letters	/	81	71	68	72	71
Loss ≥ 15 ETDRS letters	/	1	4	7	15	20

*ETDRS, Early Treatment Diabetic Retinopathy Study*.

More than two-thirds of all treated patients avoided moderate vision loss ≥ 15 ETDRS letters after 5 years of treatment. These patients preserved a statistically significant VA gain after the first year of treatment for the duration of the observation period. However, their VA progressively decreased during subsequent years of treatment (Table 2).

One-fifth of all patients lost 15 letters or more after 5 years. A statistically significant decrease in VA was observed after the fourth and fifth years of treatment. There was no significant difference in age (*p* = 0.35) and number of injections received during 5 years of treatment between the two groups (*p* = 0.70). The majority of patients maintained stable VA (variation < 15 letters) throughout the 5-year follow up. 71% of patients had stable VA at the end of the 5-year follow-up. After the first year of treatment, 19% of patients gained 15 or more letters. After 5 years of treatment, this proportion decreased to 10%. The proportion of patients that lost 15 letters or more increased gradually during the follow-up (Table 5).

All patients started treatment with either bevacizumab (97 patients) or ranibizumab (4 patients) injections. 48 patients had their anti-VEGF treatment agent switched during the 5-year follow-up. Specifically, 33 patients switched from bevacizumab to aflibercept, 13 patients from bevacizumab to ranibizumab, and 2 patients ranibizumab to aflibercept. Patients received the largest number of intravitreal injections in the first year. Thereafter, patients gradually received fewer intravitreal injections in direct correlation with the duration of treatment (Tables 2–4).

Analysis of OCT and FA imaging demonstrated the morphology of lesions for patients that lost 15 or more ETDRS letters after 5 years of treatment: subretinal fibrosis in 12 patients, atrophy in 6 patients, and active lesion in 2 patients. At the start of treatment, seven of these patients had the occult type of nAMD, seven patients had the classic type of nAMD, and six patients had retinal angiomatous proliferation.

There were no complications from administration of injections, such as endophthalmitis and retinal detachment. No serious systemic adverse events were recorded.

## Discussion

In this retrospective study, we analyzed visual outcomes of 101 eyes that were continuously treated due to treatment naive nAMD in a routine clinical practice under real-world conditions for 5 years with anti-VEGF using TER. Mean VA was essentially unchanged after 5 years of treatment. Baseline VA of patients was 60.5 (±11.3) letters and 58.6 (±18.3) letters after 5 years. We observed a gradual, albeit not statistically significant, decline of gained letters from the maximal gain after the first year toward the end of the fifth year of treatment.

The statistically significant improvement in VA was maintained for the first 3 years of treatment, but after the fourth and fifth years of treatment, VA was not significantly different from baseline values. A recent study of 54 patients treated with TER for 5 years also observed a similar trend, namely, an initial visual benefit followed by a decline in mean VA approaching baseline VA (23).

The results of the present study are in line with the results of other 3-year retrospective observational studies reported in a meta-analysis that included a systematic search of published real-world studies of intravitreal ranibizumab therapy for nAMD (20). Our observation of a mean change of +8.0, +6.3, and +5.9 letters after the first, second, and third years of treatment, respectively, is similar to the results of those studies included in the meta-analysis of Kim et al. that used TER, and observed a mean change of +8.8, +6.7, and +5.4 letters at the same time points (20). Our results are comparable with the CATT follow-up study (18), and the observational study of Gilles et al. (22). In the former, patients were treated for 2 years and spent the remaining 3 years in real-world conditions, whereas the latter analyzed data from each clinical visit of 549 patients followed for 5 years and which were collected in the Fight Retinal Blindness (FRB) database. The mean baseline VA in the CATT study was 62.2 (±13.1) (18), and in the Study of Gilles et al., 60.1 letters (±16.7) (22), compared to 60.5 (±11.3) letters in our study. After 5 years, the mean VA was 58.9 letters (±24.1) in the CATT follow-up study (18) and 59.4 letters (±20.4) in the Gilles et al. study (22), compared to 58.6 letters (±18.3) in our study. In both the CATT and Gilles et al. studies, patients received almost the same number of injections during the 5 years (18, 22). In the present study, patients had fewer visits due to the nature of treatment regimen (TER), thus reducing the treatment burden.

Patients in our study were categorized according to their baseline VA as good, intermediate, or low baseline VA. The “good baseline VA” subgroup of patients either gained fewer letters or lost more letters than the “intermediate” and “low” baseline VA subgroup of patients. Even after year 1, the “good baseline VA” subgroup did not gain letters. Pedrosa et al. (21) and the Writing Committee for the UK Age-Related Macular Degeneration EMR Users Group (24) reported similar findings, with a slight decline of mean VA over the years of treatment in their “good baseline VA” subgroup of patients. In contrast, Peden et al. reported improvement in VA in a subgroup of patients with ≥VA 20/40 (defined as “good VA group”) even after the first year of treatment and maintained the improvement till the end of the 5 years of treatment. However, studied eyes with baseline VA 20/40 or better had the smallest gains compared to intermediate and poor vision subgroups (25).

“Intermediate baseline VA” and especially “low baseline VA” subgroups gained more letters after the first year (+10.4 and +22.3) and lost less over the time of treatment. However, “good baseline VA” subgroup of patients maintained higher absolute VA at the end of the 5-year follow-up compared with the other two subgroups. The limited potential of patients with “good baseline VA” to gain more letters was termed the “ceiling effect” (26).

We observed better baseline VA in the “good final VA” subgroup of patients. The results of our study are slightly different from the study of Peden et al. (25), who used fixed-interval dosing of anti-VEGF every 4 to 8 weeks. Compared to the results of the group of patients in the study of Peden et al. (25), our group had a much higher mean baseline VA score (60.5 letters vs. 45.6 letters), a lower VA gain after the first year (+6.0 letters vs. +13.2 letters), despite a similar mean number of injections received (8.0 vs. 10.5). The difference in the VA gain can be attributed to the aforementioned “ceiling effect,” whereby eyes with good VA have smaller VA gains. Since the baseline VA was much high in our group of patients, this may have contributed to the lower VA gain observed in our patients compared to that reported by Peden et al. (25).

Although the present study did not include any specific measures of quality of life (QoL), it is well established that QoL is related to VA scores. According to Brown et al. (9), VA scores between 20/20 to 20/40 are considered to cause a greater decrement in QoL than cancer, mild stroke, or gout, whereas VA scores between 20/50 and 20/100 cause decrements that require considerable help with daily functions. VA scores lower than 20/200 are similar in terms of QoL as patients on home dialysis and bedridden due to a severe stroke. QoL is normally assessed with questionnaires. However, conclusions drawn from questionnaires could be misleading, as the questionnaires include health status measures, functional status measures, vision-specific functional status, psychological well-being, and overall QoL (27). For the purpose of the interpretation of the results of the present study, we used the recommendations of Orr et al. (28), who based their analysis on the National Eye Institute Visual Function Questionnaire-25 (NEI VFQ-25) in AMD patients. Orr et al. (28) concluded that NEI VFQ-25 scores are correlated with visual function measured by VA. VA should be sufficiently good (defined as ≥ 20/40 or ≥65 ETDRS letters) to allow normal daily activities, thus maintaining patient independence, including driving ability (4). At 5 years, 40% our group of patients had VA ≥ 65 letters, compared to the CATT follow-up study (18), where approximately 50% of patients had VA ≥ 20/40 or better. This difference can be explained by the slight difference in the age of the patients. The mean age of the patents in our study was 81.8 (±6.4) years, compared to 77.7 (±7.3) years in the CATT follow-up study (18). Mrejen et al. (23) reported that patients who receive the first injection when older were more likely to suffer from poorer VA after years of treatment.

In the present study, a high proportion (40%) of patients was assigned to the “good VA at baseline” group, and the proportion was the same after 5 years, with an increase to around 67% of patients who had good VA in the intervening years. In the FRB study, the proportion of patients with “good baseline VA” was also 40% (22), whereas in study of Pedrosa et al., it was only 15.2% (21). The main difference in the mentioned study groups was the mean baseline VA [present study: 60.5 ± 11.3 letters; Gilles et al. (22): 56.6 ± 8.7; Pedrosa et al. (21): 44.2 ± 19.0 letters]. According to Kim et al. (20), baseline VA is positively correlated with long-term visual outcome. In the FRB observational study (22), all eyes were included in the primary analysis, including patients who discontinued treatment at any point, and adjusted for the mean baseline VA of the group at the each point studied. The FRB study (22) observed that the mean baseline VA tended to increase, the longer the eyes were tracked in the system. Mean baseline VA was better in those who were treated longer; patients with better outcome are more likely to persist with treatment. Early identification of nAMD and immediate onset of treatment is therefore imperative.

In the present study, patients received a mean number of 6.1 injections per year. The highest number (8.1) was in the first year. The number of injections gradually decreased over the next 4 years (6.3, 5.9, 5.4, and 4.0, respectively). In the CATT follow-up study (18), the mean number of injections in the 3 years after the 2-year clinical trial protocol was slightly lower (15.4 ± 12.5) compared to our study from years 3 to 5 (16.2 ± 3.9). It is important to note that the lower number of injections in the CATT follow-up study (18) was associated with a greater number of visits (25.3 ± 13.3) compared to our study, in which the number of visits was the same as the number of injections. The treatment burden was therefore lower in the present study, because of the treatment regiment (TER). In the FRB study (22), patients received seven injections in the first year and five per year thereafter, whereas in study of Pedrosa et al. (21), the number of injections per year was lower. In their recent meta-analysis, Kim et al. (20) reported a significant correlation between VA outcomes and the frequency of intravitreal injections. The higher gain in mean VA after the first year of treatment was also observed in studies where patients received more intravitreal injections in the first year of treatment and in subsequent years VA outcome was better in patients who were treated intensively. In contrast, our results did not show a significant difference in the number of received intravitreal injections between patients with “good final VA” and “intermediate final VA,” which suggests that baseline VA has a more important role in predicting VA after treatment than the number of received intravitreal injections during the treatment.

Although the evidence and mechanism of geographic atrophy (GA) relating to anti-VEGF therapy remains unclear, it may be the result of increased periods of exudation requiring greater number of injections. The CATT study demonstrated that the number of anti-VEGF injections significantly correlates with development of GA. One-fifth of CATT patients developed GA within 2 years of treatment with increased risk of GA observed with monthly (every 4–5 weeks) treatment compared to PRN (29). In contrast, Peden et al. (25) reported superior visual outcomes after 5 and 7 years of follow-up using fixed-interval dosing every 4–8 weeks. They observed GA in only 3 eyes of 2 patients who lost more than 15 letters (25). On this basis, Peden et al. suggested that visual loss in other trials may be related to retinal pigment epithelium (RPE) and photoreceptor damage as a result of under treatment, rather than GA (25). In our study, using TER, we observed GA in 6 out of 20 patients that lost 15 letters or more at the end of the 5-year follow-up. Since a higher number of injections may correlate with better VA outcomes, but may increase the risk of GA development, treatment strategy may play a key role in determining the right number of injections and minimal visits. In TER, the treatment intervals (i.e., visits with injections) are adjusted according to patients’ responses to the treatment.

One of the main contributors to the development of GA is the subtype of CNV termed retinal angiomatous proliferation (RAP) (29). RAP is present in 15–20% of nAMD patients (30), resulting in GA after 2 years in 85% of patients (31). In our study population, 6 out of 20 patients that lost more than 15 letters at the end of the 5-year follow-up had RAP at baseline.

It is not uncommon for older adults to require cataract surgery, and this may have influenced on VA outcome in any patients in our study that had such surgery. Unfortunately, we did not record whether patients had cataract surgery during follow-up. Data of cataract operations in terms of proportion of patients and time of surgery performed in long-term follow-up patients are missing in almost all real-world studies. However, the evidence regarding the effect of cataract surgery on VA is equivocal. Whereas Mrejen et al. (23) reported no significant association between cataract extraction and VA and Mönestam and Lundqvist (32) observed that patients with AMD at cataract surgery had a longitudinally worse visual outcome than patients without clinical signs of AMD after 5 and 10 years of follow-up. In a shorter follow-up study by Kessel et al. (33), approximately one-fifth of AMD patients gained three lines or more of VA after cataract surgery.

A limitation of our study is its retrospective nature, and the limited set of patients. We included only patients who were followed for 5 years, required anti-VEGF treatment, and were willing to participate in the treatment. The strength of our study is that it assessed long-term visual outcomes of anti-VEGF agents in a real-world setting. All patients were treatment naïve, and all were treated with the TER regimen. They received treatment from clinical practice in a single eye center. This allowed us to have the same recommendations for starting treatment, the same method for measuring VA, and the same management criteria; however, this may limit the generalizability of the study findings.

## Conclusion

The present study demonstrates the success of the TER approach (maintaining continuous anti-VEGF therapy and adjusting injection intervals to the patients’ responses) during a 5-year follow-up in preventing severe visual loss due to nAMD. Previous studies have shown that under real-world conditions patients receive fewer injections than in clinical trials resulting in a lower initial gain in VA after the first year of therapy. This may be partly due to the treatment burden of frequent visits leading to reduced patient compliance; treatment burden of anti-VEGF therapy could be partly reduced using TER, resulting in better long-term visual outcome. To enable more nAMD patients in maintaining their vision sufficient for high level of independence, it is important, concomitant with their regular treatment, to identify nAMD and commence treatment early. Our study confirmed that patients with better baseline VA preserved good vision after long-term treatment. Further studies are warranted to determine how to maximize the proportion of eyes that will maintain good VA in long-term treatment of nAMD with anti-VEGF.

## Ethics Statement

This study was carried out in accordance with the recommendations of Committee for Medical Ethics at the Ministry of Health of the Republic of Slovenia, with written informed consent from all subjects. All subjects gave written informed consent in accordance with the Declaration of Helsinki. The protocol was approved by the Committee for Medical Ethics at the Ministry of Health of the Republic of Slovenia.

## Author Contributions

PM and PB performed this retrospective study, conducted the data analysis, and drafted the manuscript. PM designed the study and critically revised the manuscript. Both authors have read and approved the final manuscript.

## Conflict of Interest Statement

The authors declare that the research was conducted in the absence of any commercial or financial relationships that could be construed as a potential conflict of interest.
